# Can we send patients with small pneumothorax post drain removal home?

**DOI:** 10.1186/1749-8090-10-S1-A191

**Published:** 2015-12-16

**Authors:** Sanjeet SA Singh, Karim Morcos, Alan JB Kirk

**Affiliations:** 1 Golden Jubilee National Hospital, Clydebank, Dumbartonshire, G81 4DY, UK

## Background/Introduction

Pneumothorax can be a complication following chest drain removal. In thoracic surgery, access to the pleural cavity involves a pleurotomy. A chest drain is inserted to allow re-expansion of the lungs post-pleurotomy. This also prevents a tension pneumothorax. Some patients have a residual pneumothorax post-chest drain removal noted on chest radiography. Rates of pneumothorax post chest drain removal vary with figures quoted at 9.3-13.6%. The majority of these are barely perceptible or small (<1 cm from pleural line to the apex of the hemithorax). Is it safe to discharge these patients home?

## Aims/Objectives

To assess if it is safe to send patients with small pneumothorax home post chest drain removal

## Method

A retrospective observational study was done at our unit over a 6-month period. All patients had chest drains postoperatively and were discharged if there were no air leaks and the patients were stable. A repeat CXR was obtained during routine follow up in 6 weeks' time. Patients with pneumonectomies and permanent thoracostomies were excluded from the study.

## Results

There were 158 patients in the study. The mean age of the patients was 59.7 years (SD: 16.6). All patients were asymptomatic at the time of discharge and none required further intervention in other hospitals with regards to their pneumothorax. There were 29 (18.4%) patients who were discharged with small residual pneumothorax (<1 rib space) visible on CXR. At 6 weeks of follow up, 7 (4.4%) patients had visible pneumothorax on their CXR with no radiological or symptomatic worsening.

## Discussion/Conclusion

This study found that it was safe to discharge asymptomatic patients with a small pneumothorax provided they are haemodynamically stable. Our study shows that 76% of these patients will have no residual pneumothorax in 6 weeks' time and the remaining 24% will continue to be asymptomatic with no radiological worsening of their pneumothorax.

